# Dopamine D2-like receptor stimulation blocks negative feedback in visual and spatial reversal learning in the rat: behavioural and computational evidence

**DOI:** 10.1007/s00213-019-05296-y

**Published:** 2019-06-19

**Authors:** Johan Alsiö, Benjamin U. Phillips, Júlia Sala-Bayo, Simon R. O. Nilsson, Teresa C. Calafat-Pla, Arazo Rizwand, Jessica M. Plumbridge, Laura López-Cruz, Jeffrey W. Dalley, Rudolf N. Cardinal, Adam C. Mar, Trevor W. Robbins

**Affiliations:** 1 Department of Psychology and Behavioural and Clinical Neuroscience Institute, University of Cambridge, Cambridge, UK; 2 Department of Physiology, Development and Neuroscience, University of Cambridge, Cambridge, UK; 3 Neuroscience Institute, New York University Medical Center, New York, NY USA; 4 Department of Neuroscience and Physiology, School of Medicine, New York University, New York, NY USA; 5 Department of Psychiatry, University of Cambridge, Cambridge, UK; 6 Cambridgeshire & Peterborough NHS Foundation Trust, Box 190 (Liaison Psychiatry), Cambridge Biomedical Campus, Cambridge, UK

**Keywords:** Dopamine, Cognition, Dopamine D2 receptor, Rat, Dopamine D1 receptor, Reversal learning, Cognitive flexibility, Computational modelling, Hierarchical Bayesian analysis, Reinforcement learning

## Abstract

**Rationale:**

Dopamine D2-like receptors (D2R) are important drug targets in schizophrenia and Parkinson’s disease, but D2R ligands also cause cognitive inflexibility such as poor reversal learning. The specific role of D2R in reversal learning remains unclear.

**Objectives:**

We tested the hypotheses that D2R agonism impairs reversal learning by blocking negative feedback and that antagonism of D1-like receptors (D1R) impairs learning from positive feedback.

**Methods:**

Male Lister Hooded rats were trained on a novel visual reversal learning task. Performance on “probe trials”, during which the correct or incorrect stimulus was presented with a third, probabilistically rewarded (50% of trials) and therefore intermediate stimulus, revealed individual learning curves for the processes of positive and negative feedback. The effects of D2R and D1R agonists and antagonists were evaluated. A separate cohort was tested on a spatial probabilistic reversal learning (PRL) task after D2R agonism. Computational reinforcement learning modelling was applied to choice data from the PRL task to evaluate the contribution of latent factors.

**Results:**

D2R agonism with quinpirole dose-dependently impaired both visual reversal and PRL. Analysis of the probe trials on the visual task revealed a complete blockade of learning from negative feedback at the 0.25 mg/kg dose, while learning from positive feedback was intact. Estimated parameters from the model that best described the PRL choice data revealed a steep and selective decrease in learning rate from losses. D1R antagonism had a transient effect on the positive probe trials.

**Conclusions:**

D2R stimulation impairs reversal learning by blocking the impact of negative feedback.

**Electronic supplementary material:**

The online version of this article (10.1007/s00213-019-05296-y) contains supplementary material, which is available to authorized users.

## Introduction

Cognitive flexibility is required to navigate in a changing environment and requires both new associative learning and the ability to disregard rules when they become obsolete. Impairments in neuropsychological tests designed to measure such flexibility, e.g. reversal learning, are observed in psychiatric and neurological disorders including schizophrenia (Leeson et al. 2009) and Parkinson’s disease (PD; (Cools et al. 2007)). Despite this, current drug treatments often fail to remediate cognitive impairment in schizophrenia (e.g. (Leeson et al. 2009)); in the case of PD, the same drugs that restore voluntary movement by increasing dopamine (DA) tone in the dorsal striatum may even contribute to impairments in reversal learning, perhaps by “overdosing” the relatively intact ventral striatum with DA or DA D2-like receptor (D2R) agonists (Swainson et al. 2000).

Electrophysiological experiments in animals have shown that, in healthy individuals, the activity of DA neurons correlates reliably with a theoretical reward prediction error: firing rates increase in response to unexpected reward and decrease after unexpected reward omission (Schultz 2013). The causal link between this neuronal activity and reinforcement learning has been demonstrated using optogenetic approaches in rats, from the perspectives of both positive (Steinberg et al. 2013) and negative prediction errors (Chang et al. 2016). In agreement with these studies, DA activity also provides a prediction error signal during reversal learning, transiently declining in response to errors after a shift in response-outcome contingencies and increasing after unexpected rewards, as the subjects begin to interact with the previously non-rewarded, now rewarded response option (Klanker et al. 2015; Verharen et al. 2018).

At the level of the striatum, which receives the majority of midbrain DA output, D2R and D1-like receptors (D1R) are segregated between striatopallidal (indirect-pathway) and striatonigral (direct-pathway) neurons, respectively (Gerfen et al. 1990). Since D1R stimulate and D2R inhibit cAMP production, striatonigral neurons are predicted to increase cAMP and downstream signalling in response to positive prediction errors when dopamine levels transiently increase, whereas striatopallidal neurons instead respond more to negative prediction errors when dopamine levels decrease (Yapo et al. 2017). In seminal work by Frank and colleagues, reinforcement learning was altered in PD patients only after they had taken their dopaminergic medication (Frank et al. 2004): there was a selective reduction in learning from losses in a probabilistic selection task (PST), in which subjects solved two-choice visual discrimination problems either by learning to approach the positive stimuli or by learning to avoid the negative stimuli (Frank et al. 2004). A proposed explanation was that supraphysiological levels of DA block learning from negative feedback by rendering D2R-expressing striatopallidal neurons indifferent to dips in dopamine (which would impair learning from negative feedback). Hypodopaminergic states, in contrast, would not allow D1R-expressing striatonigral cells to detect DA burst firing (which would impair learning from positive feedback) (Cox et al. 2015; Frank et al. 2004). Supporting this account, variation in the *DRD2* gene was linked to learning from losses in the PST task, whereas a polymorphism in the *DARPP32* gene, intimately linked to D1R function (Calabresi et al. 2000), instead predicted learning from wins (Frank et al. 2007). Imaging experiments additionally revealed that D1R and D2R radioligand binding correlates with learning from positive and negative feedback, respectively (Cox et al. 2015). Further support for this view comes from the observation that mice lever-press for optogenetic stimulation of striatonigral neurons, whereas they avoid a lever linked to optogenetic stimulation of striatopallidal neurons (Kravitz et al. 2012). Whereas pharmacological evidence for this model of the basal ganglia is still lacking, D2Rs have been heavily implicated in reversal learning in humans (Clatworthy et al. 2009; Mehta et al. 2001), non-human primates (Groman et al. 2011; Horst et al. 2019; Lee et al. 2007), rats (Boulougouris et al. 2009) and mice (Laughlin et al. 2011; Linden et al. 2018). The evidence for D1R involvement in reversal learning, in contrast, is equivocal; e.g., systemic treatment with a D1R agonist in mice only transiently impaired visual reversal learning (Izquierdo et al. 2006), and D1R antagonism in vervet monkeys did not significantly affect reversal learning in an object reversal learning task sensitive to D2R agents (Lee et al. 2007).

To investigate the specific roles for D1R and D2R in reversal learning further, and based on recent advances in cognitive tasks for rats and mice (Markou et al. 2013; Nilsson et al. 2015; Phillips et al. 2018), we established a novel touchscreen reversal-learning paradigm for rats, in which standard two-choice visual discrimination trials (CS+ vs. CS−) were interleaved with “probe” trials, where a stimulus of intermediate valence (C_50/50_) was presented with either the CS+ or CS−. Assuming that the intermediate value of the C_50/50_ is known (by means of pre-training), subjects’ preference for the CS+ over the C_50/50_ reflects their learning from positive feedback on the standard trials, whereas negative feedback should promote a preference for the C_50/50_ over the CS−. We hypothesised that D1R antagonism would cause subjects to pay less attention to positive outcomes and thus fail to prefer the CS+ over the C_50/50_ during reversal learning, whereas subjects would become indifferent to negative feedback after D2R agonism, and hence not discriminate between the CS− and C_50/50_. A number of reference manipulations were tested, including the effect of D2R activation on computationally derived latent variables guiding behaviour in a separate group of rats tested in a serial spatial probabilistic reversal task (Bari et al. 2010).

## Materials and methods

### Compliance with ethical standards

This research has been regulated under the Animals (Scientific Procedures) Act 1986 Amendment Regulations 2012 (Project licence 70/7548) following ethical review by the University of Cambridge Animal Welfare and Ethical Review Body (AWERB).

### Subjects

Male Lister Hooded rats (Charles River, Kent, UK) were allowed to acclimatise to the animal facility under a 12 h:12 h light cycle (lights off at 7 AM) for a minimum of 7 days before any procedures began. Rats were housed in groups of 4 on wood-chip bedding in standard cages with cardboard tunnels as enrichment. When rats reached a body weight of approximately 300 g, they were food-restricted to maintain approximately 90% of their free-feeding weight trajectory (17.5–19 g of Purina rodent chow per animal and day; adjusted for reward pellet consumption during testing). Water was available ad libitum in the home cage. The experiments used a total of 124 rats (for details, see Table 1).

**Table 1:**
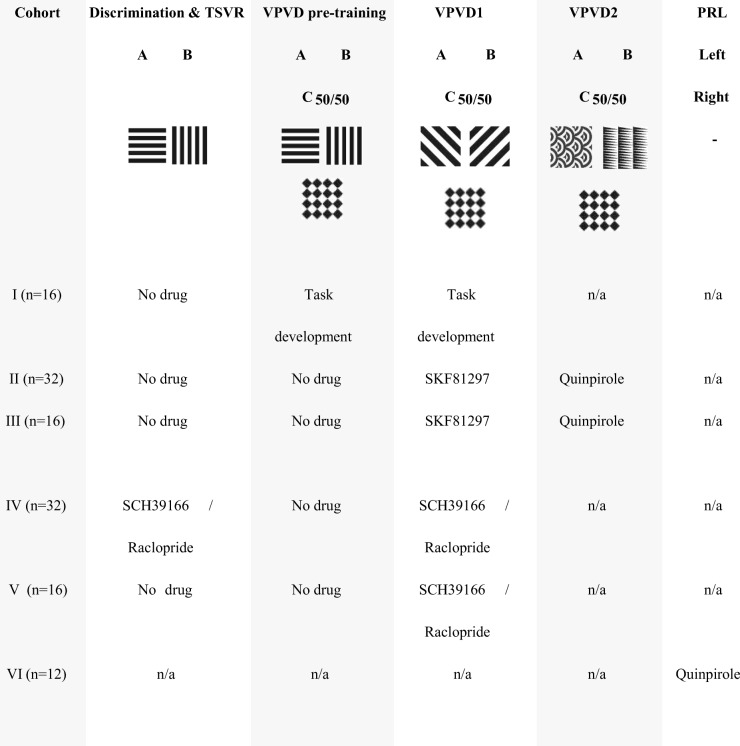
Experimental cohorts and stimuli used in the visual tasks

Stimulus-outcome contingencies were counterbalanced across drug groups. A different probe stimulus (C_50/50_) exemplar was tested in some rats in the task development experiment (not shown). *TSVR*, touchscreen serial visual reversal task; *VPVD*, valence-probe visual discrimination task; *PRL*, probabilistic reversal learning task (spatial)

### Drugs

All drugs were dissolved in saline and injected via the intraperitoneal route. Raclopride (Tocris Bioscience, Bristol, UK) was administered at 0, 0.015, 0.03, and 0.06 mg/kg, 20 min before testing. SCH39166 hydrobromide (Tocris Bioscience) was administered at 0, 0.025, 0.05, and 0.1 mg/kg, 20 min before testing. Note that SCH39166 was chosen for D1R antagonism to avoid off-target effects at the serotonin 5-HT_2C_ receptor, since activity at this receptor affects visual reversal learning (Alsiö et al. 2015). SKF81297 hydrobromide (Tocris Bioscience) was administered at 0, 0.1, and 0.25 mg/kg, 30 min before testing. (−)-Quinpirole hydrochloride (Sigma-Aldrich, St. Louis, MO, USA) was administered at 0, 0.01, 0.025, 0.1, 0.25, and 0.5 mg/kg, 60 min before testing. A broad dose range of quinpirole was chosen to allow detection of potential differential effects of canonical presynaptic and postsynaptic doses (Eilam and Szechtman 1989). No adverse reactions to repeated injections were observed in any experiments.

### Visual discrimination and reversal

#### Behavioural apparatus

Food-restricted rats were tested in 16 operant chambers (Med Associates, Georgia, VT, USA; 30 cm × 39 cm × 29 cm) placed in sound-attenuating MDF boxes with fans for the purpose of ventilation and masking external noise. A food receptacle centrally placed in one wall of the chamber was connected to an external pellet dispenser delivering 45 mg sucrose pellet (TestDiet 5TUL). A house light was located near the ceiling directly above the magazine. The wall opposite to the food receptacle was replaced by a touchscreen (Elo Touch Solutions, Inc). The chambers were controlled by in-house software (Visual Basic 2010 Express.NET, Microsoft 2010; developed by A.C.M.).

#### Pre-training

Initial touchscreen training is described in detail elsewhere (Alsiö et al. 2015); see Supplementary Table 1 for an overview. Rats were tested once daily, 5–6 days a week, throughout the experiments. Briefly, the rats were first trained to touch a large white rectangular stimulus on the screen for a sugar reward, until receiving 100 rewards within 60 min. A 5-s inter-trial interval was employed throughout all procedures. Next, the animals were required to press a medium-sized rectangle and finally a small (3 × 4 cm) “start box”, located at the bottom centre of the touchscreen. The criterion for moving on from each stage was reaching 100 responses/rewards within 60 min. During the next phase of training, pressing the start box, instead of providing pellets, lead to the presentation of a single visual stimulus on the touchscreen (“Horizontal” or “Vertical”; counterbalanced across rats and alternating between days), randomly presented left or right on the screen. Touching the stimulus lead to reward delivery whereas pressing the no-stimulus (black) side lead to a 5-s timeout, during which the house light was turned on. In order to prevent accidental contact with the screen, while at the same time promoting quick progress in the training, the stimulus was presented further down on the screen for the first sessions but moved up to approximately 7 cm height once the rat had reached > 80% correct (out of 100 trials) across 2 days. When the rat had reached > 80% on two consecutive sessions with the higher stimulus position, pre-training was complete.

#### Touchscreen visual discrimination and reversal

During the next stage of training, trials were initiated by pressing the start box as above, but rats were required to discriminate between two stimuli presented simultaneously on the screen (CS+ vs. CS−; “Horizontal” or “Vertical”; counterbalanced across rats). The animals were tested until the session they reached the running learning criterion of 24 correct in 30 trials at least once during a session (reaching criterion did not terminate the session). This visual discrimination phase normally required 1–3 days of training. A retention session was included the day after rats initially reached criterion. In addition, another retention session was then included before the stimulus-outcome contingencies were reversed. Rats were then trained on the reversed conditions until they reached the same criterion (24/30); this normally required 4–8 days of training. A single retention session was again included after rats reached criterion on reversal. See Electronic Supplementary Material for a description of the serial reversal learning task (cohort IV; cf. Table 1).

#### Valence-probe visual discrimination task with reversal

**Fig. 1:**
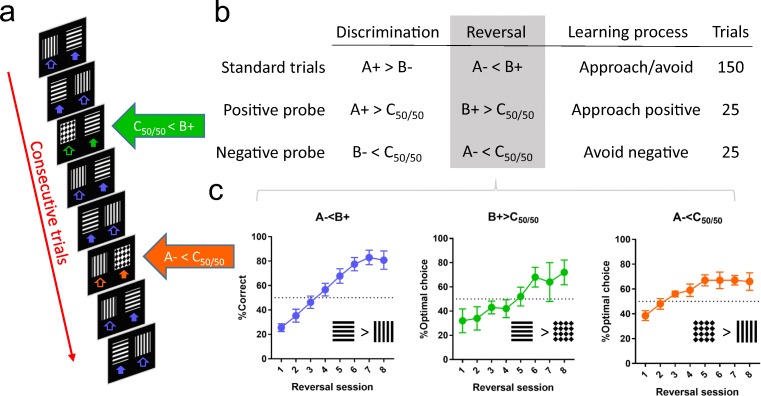
Trial structure and learning curves for the three trial types in the valence-probe visual discrimination (VPVD) reversal task. **a** Example trial sequence. **b** Regular two-choice trials during both visual discrimination (A+ > B−) and subsequent reversal learning (A− < B+) are interleaved with “probe” trials. During such trials, a third stimulus that is probabilistically linked to reward (50/50% chance of reward/no reward; C_50/50_) is presented with either the positive or the negative stimulus. c) Rats are below chance on the first day of reversal on all three trial types, indicating the influence of previously learned associations in the form of both stimulus perseveration (preference for previously rewarded stimulus, A−, over C_50/50_) and learned non-reward (avoiding previously non-rewarded stimulus, B+, when presented with C_50/50_). Choice behaviour on probe trials over the course of the reversal indicates how much the animals have learned from positive and negative feedback, respectively. Learning curves show mean ± standard error of the mean (SEM)

After the pre-training reversal was completed, the rats progressed to the valence-probe visual discrimination (VPVD) task. Here, the trial structure was kept constant but a tone was played every time a trial was rewarded and the stimulus duration was unlimited, meaning no omissions could occur. In addition, a third stimulus, probabilistically rewarded on average 50% of the time and therefore termed C_50/50_, was paired with either the CS+ or CS− on “probe” trials (Fig. 1). Initially (Experiment 1), 16 rats were trained on the VPVD reversal task (see method, below) to compare the impact of different probe stimuli (“Diamonds” and “Rings”) and frequency of probe-trial presentations (every 4 or 5 trials). For each condition, *n* = 4. Rats trained with the “Diamonds” stimulus and an average probe-trial frequency of every 4 trials (see Fig. 1a, b) displayed better performance on both visual discrimination and on reversal than other combinations (data not shown).

After optimisation, the probe stimulus was set to “Diamonds” and each of the probe trials (CS+ vs. C_50/50_ or C_50/50_ vs. CS−) was presented once every 8 trials; randomised but never on the first trial within any 8-trial bin. The rats received a maximum of 200 trials per session (i.e. 150 standard trials CS+ vs. CS−; 25 trials CS+ vs. C_50/50_; 25 trials of C_50/50_ vs. CS−). As during pre-training, both the inter-trial interval and the timeout (on non-rewarded trials) was 5 s. No omissions were allowed in the VPVD task, in order to ensure that the rats completed the probe trials. See Supplementary Fig. 1 for a comparison of the trial structure in the different behavioural tasks.

Rats were initially tested for 5 days on the same CS+ and CS− as during the pre-training reversal above (i.e. “Horizontal” vs. “Vertical”). The animals then completed a visual discrimination with a novel pair of stimuli (“Slash” vs. “Backslash”; counterbalanced across rats; CS+ and CS− is here referred to as A+ and B− during visual discrimination). Training continued for a minimum of 5 sessions, but was extended for any rat to allow them to reach 80% correct on the standard (A+ > B−) trials within the task. Next, the rats received a vehicle (saline) injection and were given a retention test session (on rare occasions, rats were given a second retention session with saline injection to achieve the 80% inclusion criterion). On the next day, rats were matched for stimulus-reward contingencies, performance on the probe trials before reversal and pre-training reversal performance, and randomly allocated to a drug group according to the experiment (Table 1). The stimulus-reward contingencies were reversed before the session and testing on the reversal phase continued for 10–14 days. The drug corresponding to each rat was administered before testing each day. Note that during reversal, the CS+ and CS− are referred to as B+ and A−, respectively. Note also that the same stimulus exemplar (i.e. “Diamonds”) was used as the probe stimulus for all rats and across each of the phases: pre-training on the VPVD task, initial visual discrimination and during the reversal phase.

As shown in Table 1, the same cohorts (II and III) received both SKF81297 and subsequently quinpirole; training during the quinpirole experiment followed the same procedure as above but rats were trained up on a new pair of stimuli (“Arcs” vs. “Triangles”; counterbalanced across rats; note that the probe-stimulus exemplar, i.e. “Diamonds”, was kept the same also throughout the second visual discrimination and reversal phase) before reversal of the new stimulus-reward contingencies (CS+ and CS− is referred to as B+ and A− during reversal). In this case, the allocation into drug groups was also balanced based on previous drug exposure. It should also be noted that the SCH39166 and raclopride cohort had been trained on a high number of serial reversals before being tested on the VPVD task (Table 1; see Electronic Supplementary Material).

#### Statistical analysis of data from the VPVD task

On the VPVD reversal task, main measures were percentage correct responses (%Correct) on the standard A− < B+ trials and performance on the probe trials across sessions (number of trials where the highest reward-probability option was chosen, i.e. B+ on B+ > C50/50 trials and C50/50 on A− < C50/50 trials; %Optimal choice). These scores were arcsine-transformed for statistical analyses, but are presented as non-transformed values in the figures. %Correct was analysed with a mixed-model ANOVA with Dose (3–6 levels) and Sessions (10 or 14 levels) as between- and within-subject factors, respectively. %Optimal choice was analysed in a mixed-model ANOVA with Dose (3–6 levels) as a between-subjects factor and Trial Type (2 levels) and Sessions (10 or 14 levels) as within-subject factors. Behaviour on the probe trials was then analysed further with two-way ANOVA for positive (B+ vs. C_50/50_) and negative probes (C- vs. C_50/50_), separately.

Data from the standard (A− < B+) trials were also divided into separate phases depending on the performance of rats during running blocks of 30 trials (Alsiö et al. 2015). Only data up to (and including) the first block of 30 trials where a rat reached criterion (24 correct) were analysed. Trials were divided into “Early”, in which the rats had less than 11 corrects in a running block of 30 trials, and “Late” if the rats scored higher than 19 correct in any block of 30 trials; all other trials were treated as “Mid”. The number of errors in each phase was calculated and square-root transformed. Repeated-measures ANOVA were then performed with two within-subject factors: phase (3 levels) and dose (4 levels). We also analysed performance on the first reversal session (Alsiö et al. 2015; Izquierdo et al. 2006). Auxiliary measures were latencies to respond to the different stimuli and latencies to collect reward (log-transformed from latencies in milliseconds and averaged across sessions; Table 2 shows the corresponding average latencies in milliseconds). For repeated-measures variables, the Greenhouse-Geisser correction was employed when prompted by significant Mauchly’s tests of sphericity. Testing of two rats was aborted and their data excluded due to computer malfunction (quinpirole 0.25 mg/kg, *n* = 1; raclopride 0.03 mg/kg, *n* = 1).

**Table 2:** Latencies to respond at the screen and to collect sucrose pellets on rewarded trials

Experiment	Response latency (ms)	Collection latency (ms)
Expt. 2 (VPVD)		
Quinpirole		
Vehicle	1066 ± 38	1529 ± 50
0.01 mg/kg	1231 ± 76	1786 ± 114
0.025 mg/kg	1261 ± 119	1916 ± 133*
0.1 mg/kg	1179 ± 59	2257 ± 104***
0.25 mg/kg	1183 ± 126	2559 ± 118***
0.5 mg/kg	1213 ± 93	2556 ± 102***
Expt. 3 (VPVD)		
SKF81297		
Vehicle	1122 ± 65	1379 ± 45
0.1 mg/kg	1279 ± 98	1436 ± 50
0.25 mg/kg	1064 ± 74	1612 ± 70**
Expt. 4 (TSVR)		
SCH39166		
Vehicle	1061 ± 71	970 ± 61
0.025 mg/kg	1137 ± 69	1126 ± 68
0.05 mg/kg	1167 ± 72	1227 ± 106
0.1 mg/kg	1190 ± 69*	1438 ± 122*
Raclopride		
Vehicle	1247 ± 91	968 ± 73
0.015 mg/kg	1171 ± 78	1013 ± 68
0.03 mg/kg	1300 ± 119	1168 ± 98*
0.06 mg/kg	1386 ± 118*	1392 ± 134*
Expt. 4 (VPVD)		
Vehicle	1047 ± 39	1344 ± 45
SCH39166 (0.05)	1209 ± 72	1508 ± 76
Raclopride (0.03)	1094 ± 66	1424 ± 41
Expt. 5 (PRL)		
Quinpirole		
Vehicle	1155 ± 179	1706 ± 72
0.025 mg/kg	2810 ± 467***	2038 ± 94**
0.1 mg/kg	4608 ± 639**	3478 ± 905*
0.25 mg/kg	5321 ± 1171**	2879 ± 243**

*VPVD*, valence-probe visual discrimination; *TSVR*, touchscreen serial visual reversal task; *PRL*, probabilistic reversal learning task. Group data are mean ± standard error of the mean (SEM), collapsed across sessions and reversal phases (Early, Mid, Late) in the TSVR and VPVD task. *p < 0.05; **p < 0.01; ***p < 0.001 vs. vehicle-treated rats in each experiment

### Serial spatial probabilistic reversal task

#### Apparatus

Testing took place in Campden Instruments (“Bussey-Saksida”) touchscreen chambers controlled by ABETII (Lafayette Instruments) and Whisker control software (Cardinal and Aitken 2010). The chambers were housed inside fibreboard boxes with fans for ventilation and to exclude noise. They were equipped with touchscreen monitors, tone generators, LED house lights and a magazine unit with light and infrared beam to detect head entries (opposite side to the touchscreen); a pellet dispenser delivered 45-mg sucrose pellets (TestDiet 5TUL). The chambers had a trapezoidal shape to guide the rats’ attention to the screen and food receptacle. We used a 5-hole “mask” to seal off most of the touchscreen; positions 2 and 4 were used throughout testing.

#### Pre-training

We adapted the established serial PRL task (Bari et al. 2010) for touchscreen chambers controlled by ABETII software written by B.U.P. See Supplementary Table 1 for an overview. Briefly, 12 rats underwent one Habituation session where ca. 30 pellets were freely available in the food tray and no task was run. Next, in a single Conditioning session, two white stimuli were presented on the screen; after the rat touched either stimuli or after 30 s had passed, the stimuli disappeared and a pellet was delivered to the food tray. Rats earned a maximum of 100 pellets in this session. If they did not complete all trials, the session terminated after 60 min. Next, during Must Touch training, no free pellets were delivered but rats could still press the stimuli for reward. These sessions terminated following 60 min or after 100 rewards had been earned, whichever occurred first. Next, animals were trained to initiate in the food magazine to begin a trial. This training stage was identical to Must Touch, except that all animals had to emit an additional nosepoke in the magazine to commence each trial. These sessions also terminated following either 60 min or after 100 pellets had been earned. Finally, all animals were trained on a Punish Incorrect stage. This was identical to the previous initiation stage except that responses at a non-target location were punished with a brief (5 s) timeout.

#### Experimental procedure

All animals were then trained on the full serial PRL procedure. This was conducted as per the final training stage, except that at the beginning of the session, one side stimulus was randomly assigned a reward probability of 80% and the other a reward probability of 20%. Following eight consecutive “correct” responses (responses to the 80% reward-probability side), the contingencies reversed so that the previously 20%-rewarded stimulus became 80%-rewarded and vice versa. These sessions terminated following either 60 min or after 200 trials had been completed. Once performance stabilised at a high level, drug administration experiments commenced. This was conducted as a within-subject Latin square, with all animals receiving all doses of quinpirole and vehicle control in a counterbalanced, pseudorandom order. For these experiments, administration sessions were always separated by a baseline session with no drug administration.

#### Statistical analyses and modelling of data from the probabilistic serial spatial reversal task

The main measures from the PRL task were the number of reversals completed per session, the win-stay probability, i.e. *P*(choose the same stimulus | rewarded on the last trial), and the lose-shift probability, i.e. *P*(choose the alternative stimulus | unrewarded on the last trial). We also analysed auxiliary measures including latency to respond and to collect rewards. Conventional statistical analyses (ANOVA) were applied to these measures.

#### Computational reinforcement learning modelling of choice data

In order to better describe the choice data from the PRL task, we applied a set of hierarchical Bayesian reinforcement learning models designed to reveal latent variables that were involved in behavioural choice. Four models were evaluated (see also Electronic Supplementary Material). The first contained parameters for reward rate (*α*_win_), which described the learning rate on rewarded trials; punishment rate (*α*_loss_), which described the learning rate on non-rewarded trials; and a softmax inverse temperature parameter (*β*), which described the degree to which choices either strongly followed stimulus value (high *β*) or were more stochastic (low *β*). The second model contained all of the above parameters and an additional side stickiness parameter (*τ*), which was designed to capture the tendency for animals to simply repeat choices at the same spatial location. The third model had a combined learning rate for rewards and punishments, a side stickiness parameter, and an inverse temperature parameter. The final model was a version of the Experience-Weighted Attraction model, which includes a parameter for the influence of previous associations (cf. (den Ouden et al. 2013)).

All models were fitted to the behavioural data by Monte Carlo sampling in Stan 2.17.2 (Stan Development Team; http://mc-stan.org) and subsequently compared by bridge sampling, which generates estimates of the marginal likelihood (Gronau et al. 2017a). This was implemented via the R package “bridgesampling” (Gronau et al. 2017b) and reveals the Bayesian posterior probability of each model given both the prior model probability and empirical data. The mean values for each parameter per group from the winning model are presented alongside the Bayesian 95% highest posterior density interval (HDI). Drug effects vs. vehicle were also sampled and evaluated as 95% HDI, which provides a robust posterior difference estimate for each parameter.

The parameters from the winning model were further evaluated by a set of simulations. Specifically, we simulated groups of rats (*n* = 40 per dose) with parameter values randomly drawn from the distribution of the estimated parameters from each drug group in the actual experiment (0; 0.025; 0.1; and 0.25 mg/kg). Each simulated rat then completed the PRL task in a virtual environment, updating the Q values and probabilities of choosing left and right (see Electronic Supplementary Material for details) depending on the four individual parameters corresponding to that rat (*α*_win_, *α*_loss_, *τ* and *β*) and the trial-by-trial feedback from the task, including probabilistically rewarded response options (80%/20%) and reversals after 8 “correct” choices in a row.

In addition, to test whether quinpirole-induced changes in individual parameters were *sufficient* to affect choice behaviour, we created sets of simulated rats (n = 40 per condition) where all but one parameter were drawn from the estimated parameter distribution for vehicle-treated rats; the last parameter was drawn from the parameter distribution of quinpirole rats. Finally, we tested the *necessity* of parameters of interest to drive the change in behaviour, by drawing e.g. *α*_loss_ from its distribution for vehicle rats and drawing the remaining three parameters (*α*_win_, *τ*, and *β*) from the relevant parameter distribution of quinpirole-treated rats.

## Results

### Experiment 1: Optimisation of the VPVD task

The VPVD task was optimised with regard to probe stimulus exemplar and probe trial frequency (Fig. 1a, b), so that rats displayed below-chance performance in probe trials for learning from both negative feedback (A− < C_50/50_) and positive feedback (B+ > C_50/50_) immediately following reversal (Fig. 1c). This indicated that the task, at these parameters, allowed us to tap into both learned non-reward (avoiding the previously negative, now positive stimulus; B+) and stimulus perseveration (approaching the previously positive, now negative stimulus; A−). All further testing was performed using these parameters.

### Experiment 2: Effects of D2R agonism with quinpirole on reversal learning

Initial inspection of the reversal data after quinpirole treatment revealed that behaviour was disrupted by the highest dose (0.5 mg/kg), with rats in this group completing fewer trials than controls (one-way ANOVA: *F*_5,41_ = 4.59; *p* = 0.002; significant reduction in trials only at 0.5 mg/kg dose; data not shown). Quinpirole treatment also dose-dependently increased latency to collect rewards (*F*_5,41_ = 13.9; *p* < 0.001; Table 2) but had no impact on latency to respond (*F*_5,41_ < 1; NS). Due to the effects on trials completed, we excluded the 0.5 mg/kg dose from further analyses.

**Fig. 2:**
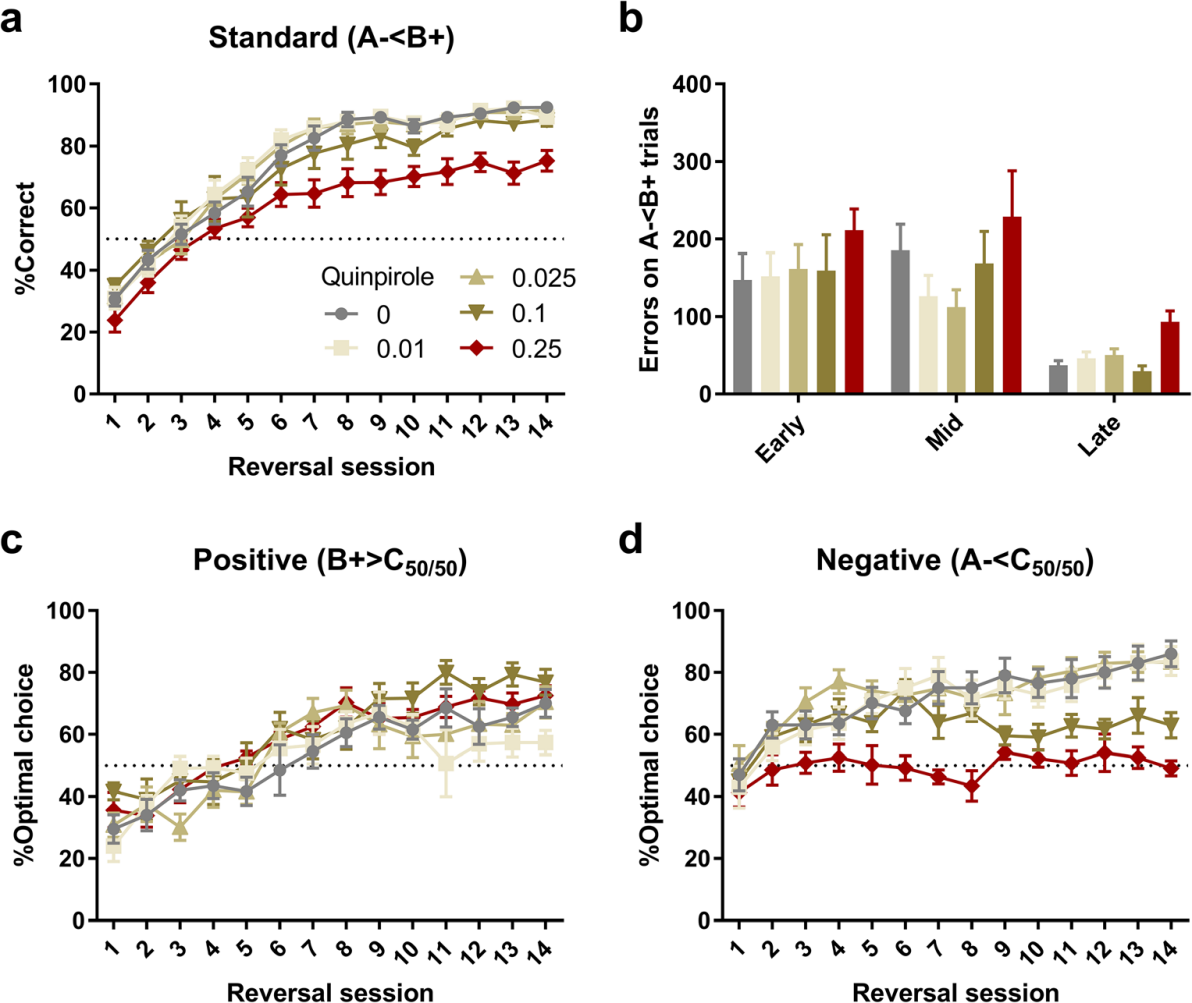
The dopamine D2-like receptor agonist quinpirole impaired visual reversal learning in the novel VPVD reversal task (*n* = 7–8 for each group). **a** Quinpirole at 0.25 mg/kg reduced correct responses on standard A− < B+ trials. **b** Quinpirole at 0.25 mg/kg increased the number of errors on standard A− < B+ trials across the learning phases (early: < 11 correct in any 30 trials; late: > 19 correct in any 30 trials, but before the criterion of 24 correct). **c** There was no effect on performance on the B+ > C_50/50_ trials, indicating intact learning from positive feedback. **d** Quinpirole dose-dependently impaired performance on the A− < C_50/50_ trials, indicating impaired learning from losses. Note that rats treated with quinpirole 0.25 mg/kg fail to improve over chance performance on negative probe trials across the 14 days of testing. Graphs show mean ± SEM for each dose and session

Quinpirole treatment impaired performance in the VPVD reversal learning task. On the standard (A− < B+) trials, there was a main effect of Dose (*F*_4,34_ = 6.83, *p* < 0.001) and a Dose × Session interaction (*F*_18.4,156_ = 2.38, *p* = 0.002). Post hoc analysis (Sidak’s method) revealed that the 0.25 mg/kg dose reduced %Correct from session 7 onwards (See Fig. 2a). The number of errors on the standard (A− < B+) trials were also analysed after trials were split into early (< 11 correct in 30 trials), mid, and late (> 19 correct in 30 trials) phases (Alsiö et al. 2015): there was a significant effect of Dose (*F*_4,34_ = 2.82; *p* = 0.04) but no Dose × Phase interaction (*F*_8,68_ < 1; NS). See Fig. 2b. Post hoc analysis (Fisher’s LSD) suggested that the 0.25 mg/kg increased overall errors compared to vehicle (*p* = 0.025).

#### Quinpirole differentially affects learning from positive and negative feedback

The quinpirole-induced impairment of reversal learning was accompanied by a selective effect on the negative probe trials (Fig. 2c, d). When analysing the %Optimal choice on the two types of probe trials in a (three-way) ANOVA, we observed a significant Dose × Session × Valence (positive vs. negative) interaction (*F*_22.6,191_ = 1.86; *p* = 0.014), indicating that the effect of Dose differed between positive and negative probe trials across sessions.

Choice performance on the positive and negative probe trials was next analysed separately. On the positive-valence probe trials (B+ > C_50/50_), there was no effect of Dose (*F*_4,34_ = 2.01; *p* = 0.11) or Dose × Session interaction (*F*_21.9,186_ = 1.22, NS). On negative-valence probe trials (A− < C_50/50_), in contrast, there was a main effect of Dose (*F*_4,34_ = 8.86, *p* < 0.001) and a significant Dose × Session interaction (*F*_30.6,260_ = 2.37, *p* < 0.001). Sidak’s post hoc comparisons revealed that the 0.25 mg/kg dose impaired performance consistently from session 7 onwards. Impairments were also observed for the 0.1 mg/kg dose from session 9 onwards.

When %Correct on the standard (A− < B+) trials of the first day was analysed separately, there was no significant main effect of Dose (*F*_4,43_ = 1.65; NS). When performance on the probe trials on the first day was examined using a two-way ANOVA with Dose as a 5-level between-subjects factor and Valence as a 2-level within-subjects factor, there were also no effect of Dose (*F*_4,34_ = 2.11; *p* = 0.10) or Dose × Valence interaction (*F*_4,34_ < 1; NS), but a significant effect of Valence (*F*_1,34_ = 11.4; *p* = 0.002).

It should be noted that the exclusion or inclusion of the 0.5 mg/kg quinpirole group did not affect the overall pattern of results. Importantly, the three-way interaction with Dose × Session × Valence remained significant also when the 0.5 mg/kg dose was included (*F*_31.1,255_ = 1.82; *p* = 0.007). In addition, previous drug history (SKF81297, Experiment 3, see below) did not significantly affect any choice measure in the quinpirole experiment (main effect and all interactions, *p* > 0.1) and the three-way interaction Dose (of quinpirole) × Session × Valence remained significant in analyses of variance where SKF81297 drug history was included as a between-subjects factor, regardless of whether all quinpirole doses were analysed together (*F*_30.7,178_ = 1.94; *p* = 0.004) or whether the highest quinpirole dose was excluded (*F*_21.8,131_ = 2.08; *p* = 0.006).

### Experiment 3: No effect of the D1R agonist SKF81297 on choice behaviour in the VPVD task

**Fig. 3:**
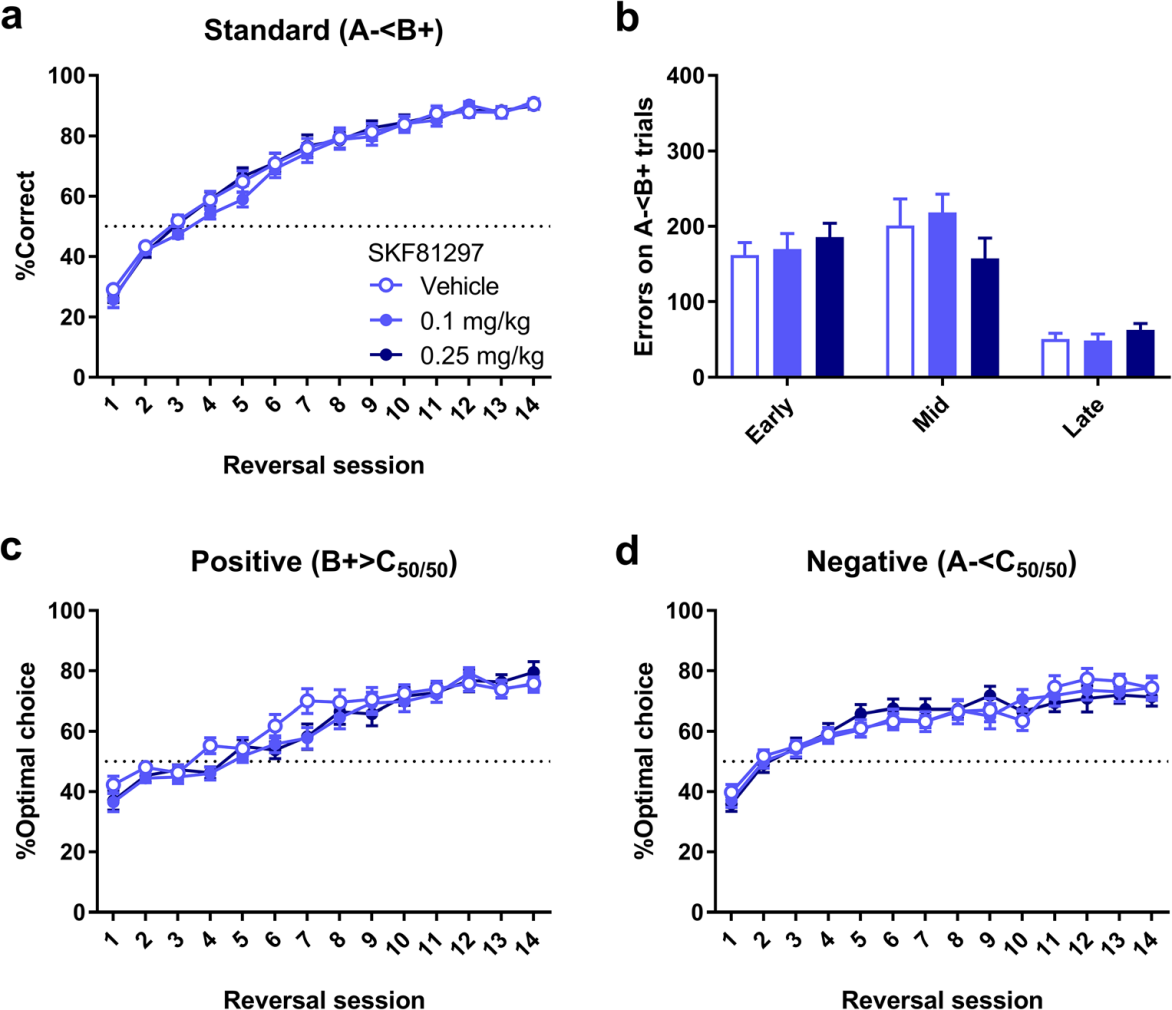
Lack of impact of the D1-receptor agonist SKF81297 on reversal learning in the VPVD task. SKF81297 did not affect learning overall at either 0.1 mg/kg (*n* = 16) or 0.25 mg/kg (n = 16) vs. vehicle (*n* = 16). **a** %Correct on standard A− < B+ trials across the 14 days of treatment. **b** Numbers of errors on standard A− < B+ trials (probe trials excluded) during early, mid, and late phases of the reversal. **c** No significant effect of SKF81297 on performance on positive probe trials. d) No effect of SKF81297 on choice behaviour on negative probe trials. Graphs show mean ± SEM for each dose and session

The D1R agonist SKF81297 had no appreciable effects on reversal learning overall (Fig. 3a). In a two-way ANOVA, there was no main effect of Dose (*F*_2,45_ < 1; NS) on performance on the standard (A− < B+) trials, and no Dose × Session interaction (*F*_8.39,189_ < 1; NS). Performance on the standard (A− < B+) trials were next split into early, mid, and late phases, as above, and analysed in a two-way ANOVA. There was no significant effect of Dose (*F*_2,45_ < 1; NS) or a Dose × Phase interaction (*F*_3.11,69.9_ = 1.95; *p* = 0.13) on errors in the different phases (Fig. 3b).

The combined effects of Dose and Session on %Optimal choice were investigated in a three-way ANOVA (Fig. 3c, d). There was no Dose × Session × Valence interaction (*F*_11.1,251_ = 1.25, NS), and also no significant main effect of Dose in the full model (*F*_2,45_ < 1; NS). Despite the lack of a significant three-way interaction, the performance on the positive and negative probe trials was next analysed separately for potential trends in the data. On positive probe trials, there was no significant effect of Dose (*F*_2,45_ < 1; NS) and no significant Dose × Session interaction (*F*_12.6,283_ = 1.14; NS). On negative probe trials, there was similarly no effect of Dose (*F*_2,45_ < 1; NS) and no significant Dose × Session interaction (*F*_12.9,290_ < 1; NS).

SKF81297 did not affect latencies to respond at the stimuli (*F*_2,45_ = 1.80; NS) but did have an impact on reward collection latencies (*F*_2,45_ = 4.41; *p* = 0.018); post hoc analyses revealed that the 0.25 mg/kg dose increased the latency to collect the reward (Table 2).

### Experiment 4: Effects of D1R and D2R antagonism on reversal learning

In a preliminary experiment, we evaluated a range of doses for SCH39166 (0; 0.025; 0.05; 0.1 mg/kg) and raclopride (0; 0.015; 0.03; 0.06 mg/kg) on the touchscreen serial visual reversal task (Electronic Supplementary Material). Doses used for the VPVD task were based on a lack of effect on response latencies; there were also no effects of SCH39166 or raclopride on errors on the serial visual task (Supplementary Fig. 2).

**Fig. 4:**
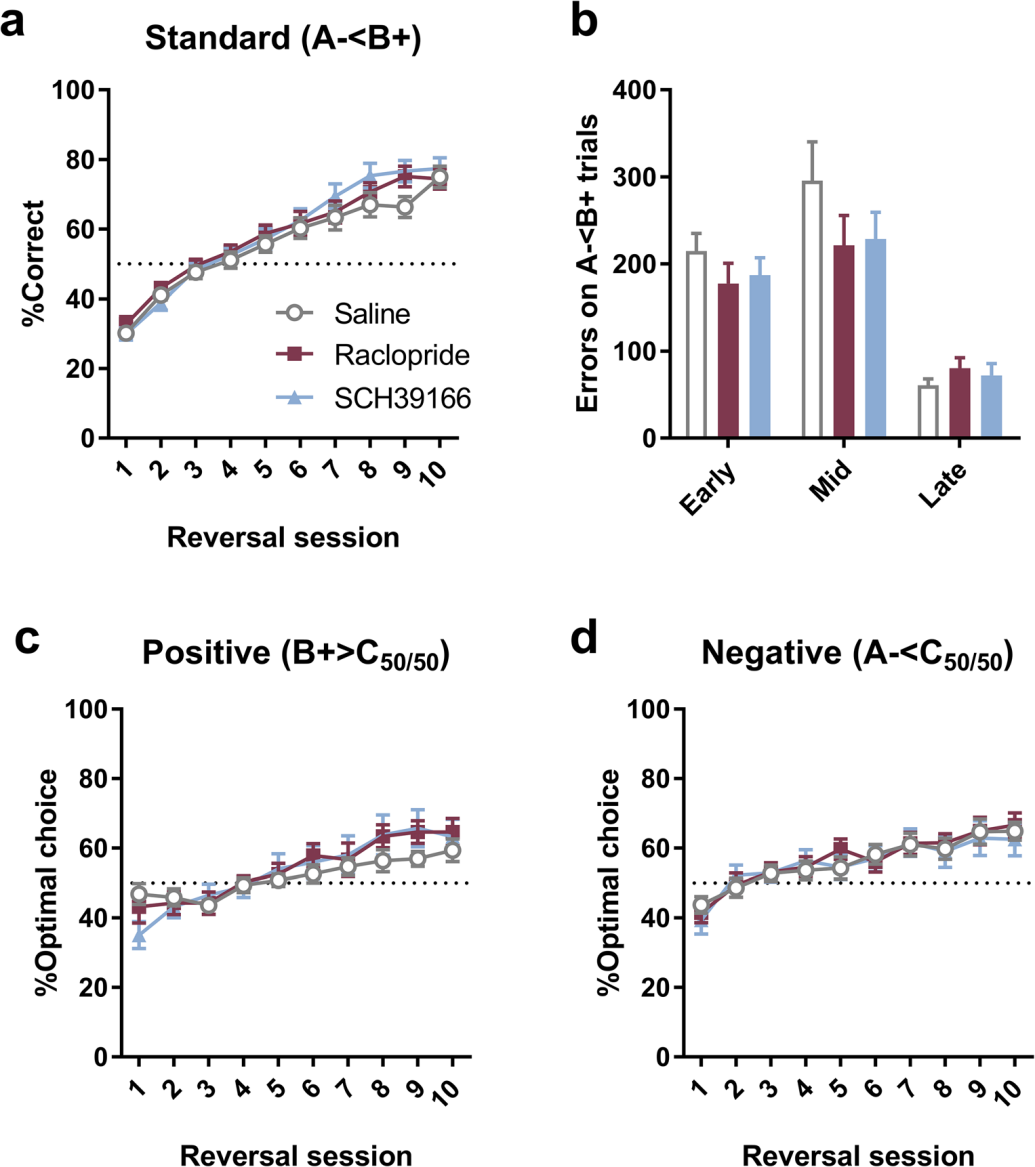
Performance on the VPVD reversal task after dopamine receptor antagonism. D2-like receptor antagonism (raclopride; 0.03 mg/kg; *n* = 13) and D1-like receptor antagonism (SCH39166; 0.05 mg/kg, *n* = 14) had no significant effect versus vehicle treatment (saline; *n* = 20). **a** %Correct over each of 10 sessions. **b** No effect on number of errors on standard A− < B+ trials committed during each of three learning phases. **c**, **d** Performance on probe trials. Raclopride and SCH39166 did not significantly affect learning overall on either positive (B+ > C_50/50_) or negative (A− < C_50/50_) trials. The graphs show mean ± SEM for each dose and session

In the VPVD reversal task, no significant overall effects were detected after either D1R antagonism (SCH39166) or D2R antagonism (raclopride) compared to vehicle-treated rats (Fig. 4a). Percentage correct on standard (A− < B+) trials was investigated with a two-way ANOVA with Treatment (vehicle, SCH39166, or raclopride) as a between-subjects factor and Session as a within-subjects factor (10 levels). We observed no effect of Treatment (*F*_2,44_ < 1; NS) and no Treatment × Session interaction (*F*_5.56,122_ = 1.29; NS). We also analysed the performance on the A− < B+ trials from the perspective of different phases in reversal learning (early, mid and late; Fig. 4b). There was no main effect of Treatment (*F*_2,45_ < 1; NS) and no Treatment × Phase interaction (*F*_3.17,71.3_ < 1; NS) in a two-way ANOVA.

#### Transient effect of D1R antagonism on positive feedback

Performance on the probe trials was investigated next (Fig. 4c, d). We observed no significant three-way Treatment × Session × Valence interaction (*F*_10.1,221_ < 1; NS) and no main effect of Treatment (*F*_2,44_ < 1, NS) in the full model. To test the a priori hypothesis that dopamine D1-receptor antagonism impairs learning from positive feedback, we next investigated the effects of SCH39166 and raclopride on positive and negative probe trials separately (despite a lack of a significant three-way interaction). On positive probe trials, there was no main effect of Treatment (*F*_2,44_ < 1; NS) and no Treatment × Session (*F*_9.88,217_ = 1.49; *p* = 0.15). On negative probe trials, there was similarly no effect of Treatment (*F*_2,44_ < 1; NS) and no significant Treatment × Session interaction (*F*_11.2,247_ < 1; NS).

We also tested the hypothesis that D1- and D2-receptor antagonism preferentially affects early reversal learning. In a one-way ANOVA with Treatment as the between-subject factor (3 levels), we found no significant effect on %Correct on the standard (A− < B+) trials on the first day of reversal (*F*_2,45_ < 1; NS). In a two-way ANOVA with Treatment as a between-subjects factor and Valence as a within-subjects factor, we observed a significant main effect of Treatment (*F*_2,45_ = 5.45; *p* = 0.008) but no effect of Valence (*F*_1,45_ < 1*;* NS) and no Treatment × Valence interaction (*F*_2,45_ < 1; NS). As we had a priori expectations about differential effects across learning from positive and negative feedback, we investigated simple main effects despite the lack of a significant two-way interaction. Analysis of simple main effects using Fisher’s LSD comparisons revealed a significant effect of SCH39166 on the positive probe trials on the first day (*p* = 0.033); all other comparisons were non-significant.

In agreement with our preliminary experiments, there was no effect of SCH39166 or raclopride dose on latency to respond in a one-way ANOVA (*F*_2,45_ = 1.83; NS); in a separate ANOVA, there was also no significant effect on latency to collect the reward (*F*_2,45_ = 1.73; NS). See Table 2.

### Experiment 5: Effects on D2R agonism on a spatial PRL task

**Fig. 5:**
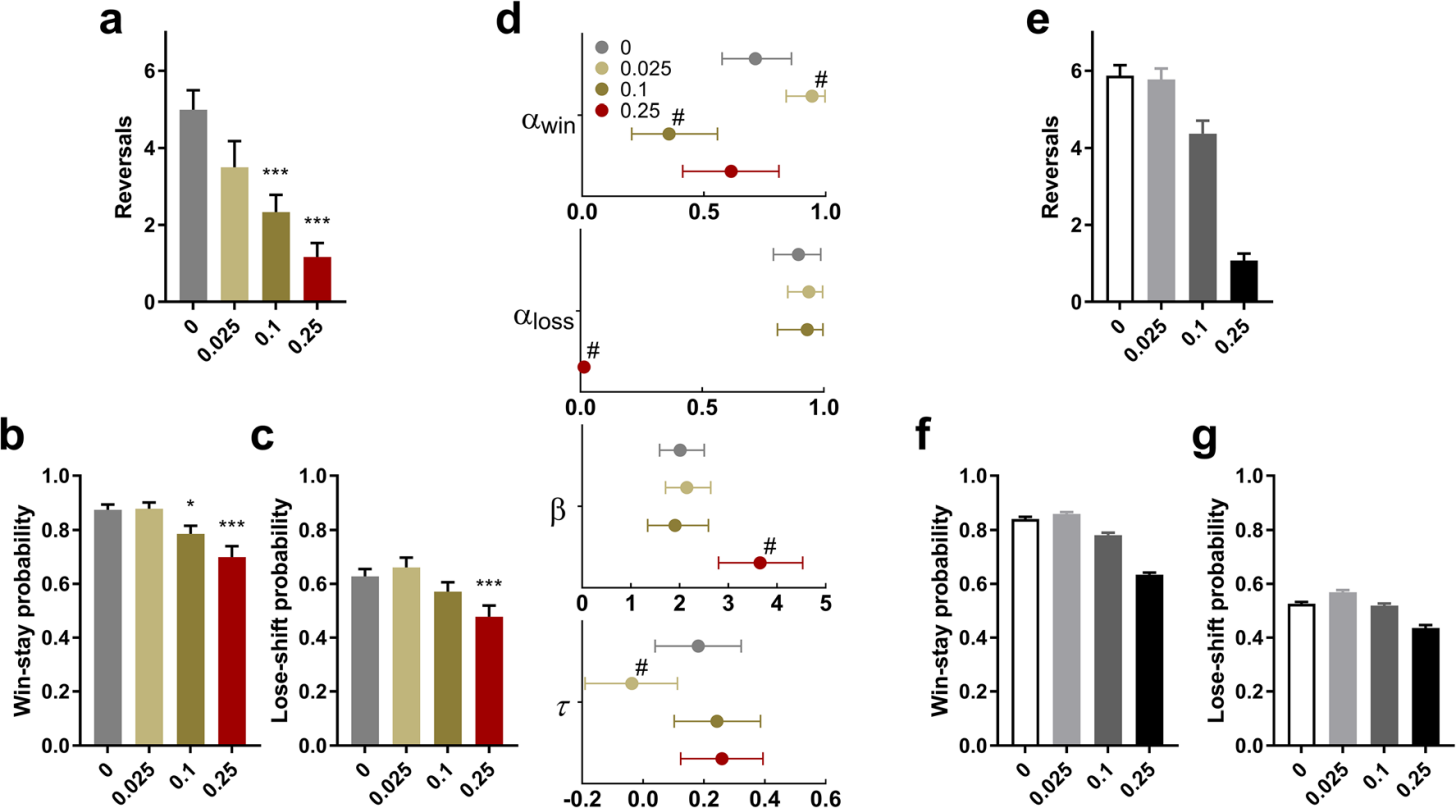
The D2R agonist quinpirole impaired reversal learning in the PRL task. **a** Dose-dependent decrease in the number of reversals completed after quinpirole injections (mean ± SEM). **b**, **c** Quinpirole impaired both win-stay (**b**) and lose-shift (**c**) performance (mean ± SEM). **d** 95% highest posterior density intervals (HDI) for parameters estimated by hierarchical Bayesian analysis of trial-by-trial choice data from the PRL task. The best model was a reinforcement learning account with separate learning rates for wins (*α*_win_) and losses (*α*_loss_), inverse temperature (*β*), and side stickiness (*τ*). Quinpirole 0.25 mg/kg impaired learning rate after losses (*α*_loss_) without affecting learning rate for wins (*α*_win_). This dose also increased the inverse temperature. **e**–**g** Using the winning model, we simulated rats performing the reversal task in silico and updated their expected outcomes (Q values; see Supplementary Online Material) on a trial-by-trial basis using feedback such as probabilistically rewarded responses and reversals after 8 correct responses in a row. For each simulated group (*n* = 40/dose; graphs show mean ± SEM), parameter values were randomly drawn from the estimated distribution of the actual rats at the corresponding dose. **e** Dose-dependent decrease in the number of reversals in the simulation. **f**, **g** Win-stay and lose-shift analysis of choice data from the simulated rats reveals that the behaviour of the actual rats is recovered by the winning model. *p < 0.05 vs. vehicle; ***p < 0.001 vs vehicle; ^#^the 95% HDI for the difference score (vs. vehicle) excluded zero, i.e. there is a > 95% probability that the drug effect was non-zero

Quinpirole impaired learning and increased latencies to respond and collect rewards on the PRL (Fig. 5a–c; Table 2). In a one-way ANOVA, quinpirole dose-dependently decreased the number of reversals completed (*F*_3,33_ = 13.4; *p* < 0.001); post hoc analyses revealed that this effect was significant at both 0.1 mg/kg and 0.25 mg/kg quinpirole (Fig. 5a). In a two-way ANOVA with Trial Type (win-stay, lose-shift) and Dose (4 levels) as within-subject factors, there was a main effect of Dose (*F*_3,33_ = 13.8; *p* < 0.001) but no Dose × Trial Type interaction (*F*_3,33_ < 1; NS). We nevertheless investigated the trial types separately, as these can be speculated to relate to learning from positive and negative feedback, respectively (but see the computational analysis below, for more robust measures). Quinpirole had a significant impact on win-stay probability (*F*_2.1,23.1_ = 11.0; *p* < 0.001); post hoc analyses revealed significantly decreased values for both 0.1 mg/kg and 0.25 mg/kg (Fig. 5b). There was also a significant effect of quinpirole on lose-shift performance (*F*_3,33_ = 6.41; *p* = 0.002); this effect was driven by the 0.25 mg/kg treatment (Fig. 5c). Furthermore, quinpirole increased latencies both to respond on the screen (one-way ANOVA; *F*_1.71,17.1_ = 16.5; *p <* 0.001) and to collect the sugar pellet (one-way ANOVA; *F*_1.51,15.1_ = 9.25; *p* = 0.004) on rewarded trials. Post hoc analyses showed that all doses of quinpirole increased both latency to respond and collect rewards in the PRL (Table 2).

#### Hierarchical Bayesian modelling of PRL choice data

We next used hierarchical Bayesian analysis of reinforcement learning to sample latent variables influencing behaviour in the probabilistic spatial serial reversal task. Four different models were compared; the best description of the choice data (via bridge-sampled maximum likelihoods) was found to be the model containing separate learning rates for wins (*α*_win_) and losses (*α*_loss_), a softmax inverse temperature parameter (*β*) and a side stickiness parameter (*τ*). For details, see Electronic Supplementary Material.

We explored the effect of drug treatment on the posterior distributions of the group means for *α*_win_, *α*_loss_, *β*, and *τ* (Fig. 5d). For *α*_win_ (learning rate for wins), 0.025 mg/kg increased this measure relative to the vehicle condition (0 ∉ 95% HDI for group differences). Conversely, 0.1 mg/kg decreased *α*_win_ relative to vehicle treatment (0 ∉ 95% HDI for group differences). In contrast, the 0.25 mg/kg dose did not affect *α*_win_. There was a sharp decrease in *α*_loss_ (learning rate for losses) at the highest dose of quinpirole (0.25 mg/kg) compared to the vehicle condition (0 ∉ 95% HDI for group differences). No other doses exerted an effect on this parameter. Additionally, high-dose (0.25 mg/kg) quinpirole increased *β* (inverse temperature) relative to vehicle treatment (0 ∉ 95% HDI for group differences), whereas other doses did not affect this parameter. Finally, low-dose quinpirole (0.025 mg/kg) decreased *τ* (side stickiness) relative to vehicle treatment (0 ∉ 95% HDI for group differences), but no other differences in which the 95% HDI did not contain 0 were detected on this measure.

#### Simulated task performance: posterior predictive check and role of individual model parameters in driving reversal impairment

To interrogate the validity of the winning model and better understand the contribution of changes in reinforcement learning parameters to overall performance, we simulated the choice behaviour of agents on the serial PRL task based on the extracted parameters in the winning model. The simulations closely matched the raw data on the main task measures, with dose-dependent trends on reversals completed (Fig. 5e) and win-stay and lose-shift proportions (Fig. 5f, g).

**Fig. 6:**
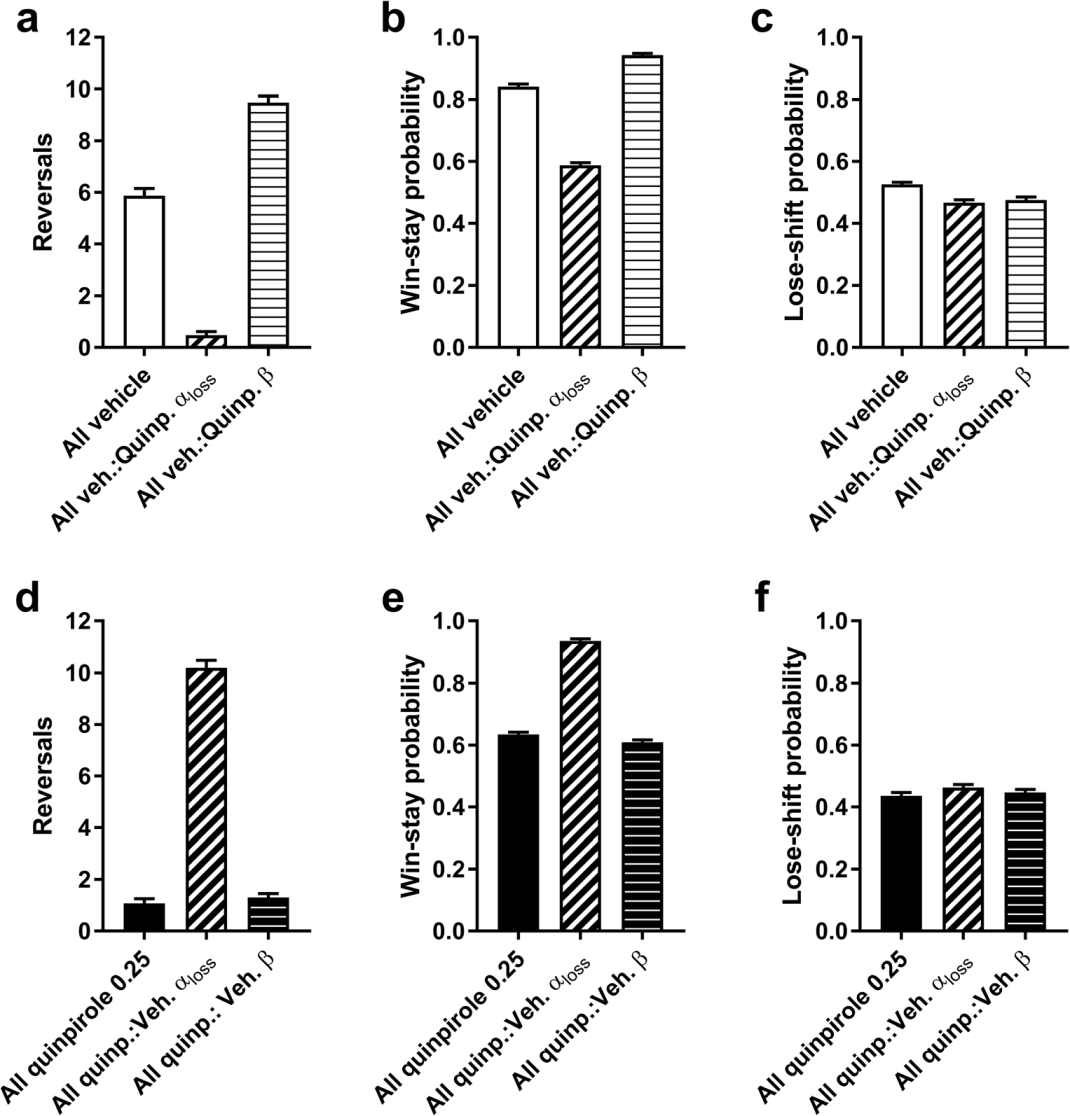
Simulations reveal that the effects of quinpirole 0.25 mg/kg on the *α*_loss_ parameter is sufficient and necessary to drive reversal learning impairment. **a** Test for sufficiency of the *α*_loss_ parameter to drive reversal impairment. In simulated vehicle-treated rats (“All vehicle”; see Fig. 5), impaired reversal learning is observed when the *α*_loss_ parameter is replaced by values drawn from the estimated distribution of quinpirole 0.25 mg/kg rats (“All veh.:Quinp. *α*_loss_”). In contrast, replacing the *α*_loss_ parameter with *β* drawn from the distribution of quinpirole 0.25 mg/kg rats (“All veh.: Quinp. *β*”) actually improves simulated performance, as measured by reversals completed. **b** The same pattern is observed on win-stay probabilities, where “All veh.:Quinp. *α*_loss_” rats perform worse than both “All vehicle” and “All veh.: Quinp. *β* rats”. **c** On lose-shift probabilities, simulated vehicle-treated rats with either the *α*_loss_ or the *β* drawn from the distribution of quinpirole 0.25 mg/kg rats display impaired performance on the virtual task. **d** Test for necessity of the *α*_loss_ parameter to drive reversal impairment. Simulated 0.25 mg/kg quinpirole rats (“All quinpirole 0.25”) perform the virtual task poorly (cf. Figure 5). Replacing the *α*_loss_ of these simulated rats with the values drawn from the distribution of vehicle-treated rats (“All quinp.:Veh. *α*_loss_”) restores performance on the virtual task. In contrast, replacing only the *β* with values drawn from vehicle rats (“All quinp.:Veh. *β*”) does not improve performance as measured by the number of reversals. **e** “All quinp.:Veh. *α*_loss_” rats outperform both “All quinpirole 0.25” and “All quinp.:Veh. *β*” on the win-stay probabilities. **f** On lose-shift probabilities, there were no differences between “All quinpirole 0.25” rats and the “All quinp.:Veh. *α*_loss_” and “All quinpirole 0.25” groups. Graphs show mean ± SEM; *n* = 40 for each condition

Next, we reasoned that the changes in task performance detected following high-dose quinpirole administration could be attributed to either the increase in *β* or the reduction in *α*_loss._ To distinguish between these competing explanations, we carried out simulations of task performance where virtual agents would be allocated one extracted parameter from the quinpirole group (0.25 mg/kg) and maintaining all other parameters at vehicle levels (Clarke et al. 2014). This revealed that simulating performance with only the high-dose *α*_loss_ values was sufficient to closely replicate the reversal (Fig. 6a) and win-stay/lose-shift values (Fig. 6b, c) from the high-dose quinpirole group; in contrast, agents with the high-dose *β* values were, in fact, better than simulated vehicle rats on both reversals completed and win-stay probability (Fig. 6a–c).

Having established that the change in high-dose *α*_loss_ was *sufficient* to replicate the impairments in task performance, we also tested whether it was *necessary* to bring about this pattern of results (Fig. 6d–f). To achieve this, we carried out further simulations in which one parameter was set to vehicle levels and the others were maintained at high-dose quinpirole levels. This revealed that the reduction in *α*_loss_ was necessary to recapitulate the impairments that were observed in rats treated with high-dose quinpirole. This effect was observed in number of reversals completed (Fig. 6d) and in win-stay probability (Fig. 6e), but less so in the lose-shift performance (Fig. 6f).

## Discussion

We introduce a novel touchscreen paradigm that enables investigation of how positive and negative feedback shape behaviour in visual reversal learning. This is achieved by intermittently inserted probe trials that inform how subjects track specific stimulus values across changing stimulus-reward contingencies. In order to exploit the translational potential of the touchscreen methodology, we tested two hypotheses pertaining to dopaminergic influences on reversal learning, based in part on the reinforcement learning data from the probabilistic selection task in humans (Frank et al. 2004, 2007). We found strong evidence for the hypothesis that D2R agonism would impair reversal learning by selectively blocking learning from losses. In contrast, we found only weak evidence for an impact of D1R antagonism on learning from positive feedback in the visual task. The selective impairment in learning from negative feedback after D2R agonism was substantiated using computational analysis from another, spatial probabilistic reversal task.

### D2R stimulation selectively blunts learning from losses in reversal learning

Quinpirole treatment severely impaired reversal learning at the higher end of the dose range in both deterministic visual and probabilistic spatial tasks. Whereas these observations are supported by previous studies where D2R agonism impaired reversal learning in rats (Boulougouris et al. 2009), non-human primates (Smith et al. 1999) and healthy volunteers (Mehta et al. 2001), as well as by experiments linking variation in the *DRD2* gene to reversal learning (Smith et al. 1999), our data extend such findings by showing a dose-dependent and highly selective effect of quinpirole on learning from negative feedback in the novel VPVD reversal task for rats. Even after 14 days of training, animals receiving high doses of quinpirole could not discriminate between the non-rewarded response option (A−) and the probabilistically reinforced probe stimulus; at this stage, the rats had learned to choose the B+ on the positive probe trials with high accuracy. Under the assumption that quinpirole at the relevant doses (≥ 0.1 mg/kg) act on postsynaptic D2R in the striatum to inhibit the activity of striatopallidal neurons, our data provide support for the view that the indirect pathway of the basal ganglia predominantly contributes to learning from negative feedback, or avoidance learning (Cox et al. 2015; Frank et al. 2004). Whereas this interpretation needs to be confirmed in future studies directly manipulating striatopallidal neurons or D2R within the striatum, the present study adds receptor specificity to previous pharmacological data linking hyperdopaminergic states in the rat to impaired learning from losses in reversal learning (Verharen et al. 2018). In addition, the strong link between D2R and learning from losses suggests a psychological mechanism behind enhanced sensitivity to negative feedback and concomitant risk aversion after D2R pharmacology and interrogation of D2R-positive neurons in the nucleus accumbens in the study by Zalocusky et al. (Zalocusky et al. 2016).

Quinpirole also impaired performance in the PRL task, where the number of reversals passed, as well as win-stay and lose-shift behaviour, were all reduced in a dose-dependent manner. Strikingly, parameter estimation using hierarchical Bayesian analysis revealed a complete blockade of learning from negative feedback (*α*_loss_) at the 0.25 mg/kg dose. In contrast, there was no effect on learning from positive feedback (*α*_win_) at this dose, although lower doses tended to either enhance (0.025 mg/kg) or reduce (0.1 mg/kg) this parameter. The 0.25 mg/kg dose also increased the inverse temperature parameter, *β*. Elevated *β* indicates higher reinforcement sensitivity (less randomness or “exploring”), suggesting that rats on high-dose quinpirole were more guided by the expected outcomes of responses; i.e., that rats would obey the trial-by-trial Q values and “exploit” rather than “explore”. Hence, impairments in reversal learning could logically be driven by either decreased *α*_loss_ or increased *β* (or their combination). (Note that exploiting the expected value after a contingency reversal leads to perseveration on the task, as long as the *Q* value for the previously correct, now incorrect choice remains high.)

We used simulations to estimate the causal contribution to reversal-learning deficits of the parameters that were affected by 0.25 mg/kg quinpirole, i.e. *α*_loss_ and *β*. We found that simulated “vehicle” rats, whose values for the *α*_loss_ parameter were replaced with values drawn from the “0.25 mg/kg quinpirole” group, were as poor on the virtual task as were the simulated “0.25 mg/kg” rats. In addition, we found that simulated “quinpirole 0.25” mg/kg rats, whose values for the *α*_loss_ parameter was replaced with values drawn from the “vehicle” group, were significantly better than “0.25 mg/kg” rats. No such effects were observed for *β*. This suggests that the steep reduction in the *α*_loss_ parameter is both sufficient and necessary for the impairments observed after 0.25 mg/kg quinpirole treatment. Taken together, the quinpirole data from both tasks reveal an apparently complete blockade in updating behaviour in response to losses in reversal learning, manifested as an inability to learn to avoid the CS− in the visual setting and an *α*_loss_ approaching zero in the PRL.

### D2R stimulation does not affect learning from positive feedback in the VPVD task

Quinpirole had no effect on performance on the positive probe trials in the visual setting, and a less consistent effect on learning rate for wins in the spatial PRL task. Whereas a lack of effect of D2R agonism on learning from positive feedback in the visual task is in agreement with our hypothesis and the reinforcement learning literature (Cox et al. 2015), the finding is at odds with other results. For instance, studies by Groman and colleagues showed that the D2R binding in the striatum of vervet monkeys correlated with reactivity to positive feedback in a reversal task (Groman et al. 2014, 2011). However, the method used to measure learning from positive feedback in those studies was win-stay behaviour. In the present PRL data, we observed a strong reduction in win-stay probability in the 0.25 mg/kg group, but this did not, however, translate to altered *α*_win_. It is tempting to suggest that computational modelling provides a more nuanced account of behaviour than does the win-stay analysis, by taking factors other than the immediate response to reward into account when interpreting subjects’ choices. In agreement with this, Verharen and colleagues recently found reduced win-stay probability in a spatial deterministic reversal task after cocaine and amphetamine pre-treatment, but a selective effect on *α*_loss_ in the computational analysis (Verharen et al. 2018). Evidence in human volunteers describes how D2R antagonism, which had no effect in our visual task, affected choice performance (inverse temperature, *β*) but not reinforcement learning per se (learning rate, *α*) in a task where learning was guided by rewards, whereas no such effect was observed when learning was driven by negative reinforcement (Eisenegger et al. 2014; Pessiglione et al. 2006).

### Low doses of quinpirole affect collection latencies not choice performance

Lower doses of quinpirole (≤ 0.025 mg/kg), which may act predominantly on presynaptic receptors (Ford 2014), failed to alter choice performance in the visual paradigm. Nevertheless, reward collection latencies were significantly slower at this dose and upwards, indicating blunted motivation for the reward. This shows that the 0.025 mg/kg dose was biologically active and suggests that there is a dissociation between lower and higher quinpirole doses on motivational and cognitive aspects of the task. Speculatively, quinpirole reduces motivation at lower doses by acting on presynaptic autoreceptors to inhibit activity in midbrain dopamine neurons. In line with this view, quinpirole microinfusions into the ventral tegmental area have been reported to reduce motivation for sucrose and ethanol (Hodge et al. 1993), and RNA interference of D2 receptors (hence, reduced D2 autoreceptor activity) in the ventral tegmental area increases motivation for sucrose and drug reward (de Jong et al. 2015). Such presynaptic effects appear insufficient to change reinforcement learning, both after D2R silencing (de Jong et al. 2015) and in the present data set.

### D1R and positive feedback

Dopamine D1R antagonism had no overall effect on reversal learning, but caused a transient decrease in performance on the positive probe trials, indicating impaired learning on the very first reversal session. Thus, our hypothesis, that learning from positive feedback would be selectively impaired by D1R antagonism, gained no conclusive support. Nevertheless, our observation of no overall impairment of D1R antagonism on reversal learning is in agreement with a lack of effect of systemic SCH23390 treatment on reversal learning in vervet monkeys (Lee et al. 2007). Similarly, D1R agonism had no effect on any of the main measures in this experiment; this is in apparent contradiction with a previous reversal-learning experiment where a transient effect on the first sessions was observed after injections of the same drug in mice (Izquierdo et al. 2006). It is conceivable that the performance-impairing effects of D1R agonism and antagonism on neurons in the striatum are confounded by enhancing effects at other sites (e.g. in the cortex, although see (Calaminus and Hauber 2008)). In addition, our prediction of performance-impairing effects of D1R antagonists was based on reports from discrimination learning tasks (e.g. (Frank et al. 2007; Kravitz et al. 2012)), and we acknowledge that D1R may play additional or opposing roles during reversal learning, where previous associations have to be overcome for successful task performance. Such opposing effects may be dose-dependent, and potentially unmasked at alternative dose intervals than those used in the current set of experiments. Taken together, future studies should focus on the effects of D1R manipulations on, e.g. initial visual discrimination learning or investigate the effects of local micro-infusion of D1R agents into brain areas of interest in rats performing reversal-learning tasks. Furthermore, the failure of any dopamine agents tested here to affect learning to approach the positive stimulus warrants the investigation of drugs acting on other neuromodulators or neurotransmitter systems to identify the mechanisms of positive feedback in reversal learning.

### Strengths and weaknesses of the VPVD task

The design of the VPVD task comes with inherent strengths and weaknesses. The main strength is that the probe trials, during which rats choose between the intermediate stimulus and either the positive or negative response options, allow us to track stimulus preferences across the length of the reversal phase (from initial performance, which is worse than chance, to an asymptote above chance). This approach revealed that quinpirole treatment selectively affects choice behaviour on the A− vs. C_50/50_ trials and that this effect is most apparent during later sessions of reversal learning. Our paradigm thus provides a novel method for studying stimulus perseveration (inhibiting the response at the previously rewarded stimulus, A−) and learned non-reward (approaching a previously non-rewarded stimulus, B+) in visual reversal learning, and builds upon previous work addressing these phenomena using e.g. three response options or replacement of one of the stimuli with a novel stimulus during the reversal phase (Alsiö et al. 2015; Clarke et al. 2007; Piantadosi et al. 2019); for a review, see (Nilsson et al. 2015). A selective blockade of learning from negative feedback, as reported here, is in agreement with previous findings of quinpirole-induced delays in overcoming perseverative responding at the previously correct response option in spatial reversal learning (Boulougouris et al. 2009). However, our data (see Fig. 2) strongly suggest that impaired learning from losses does not equate to poor performance preferentially during the early phase of visual discrimination reversal.

An alternative approach to studying learning from positive and negative feedback is used in the probabilistic selection task (Frank et al. 2004), where subjects initially learn the value of stimulus pairs without any probe trials, and new pairings are presented after learning has already taken place, in order to explore whether subjects have learned from positive or negative feedback. In the context of two-choice visual reversal learning (A− vs. B+), this approach can be implemented as probe sessions with novel pairings (A− vs. C_50/50_ and B+ vs. C_50/50_) after a pre-defined number of trials or sessions (e.g. every five sessions for rodents). Whereas this would not have allowed us to follow the learning on a session-by-session basis, as the probe trials did in our design, the extent to which rats had learned from positive or negative feedback could have been evaluated in the drug-free state, separating the effects of drugs on choice from the effects of drugs on learning. In addition, although we here interpret choices on the probe trials as reflecting the extent to which the rats have learned about stimuli A and B during the standard trials, it is conceivable that the rats are solving the three different pairings (A vs. B; A vs. C_50/50_; B vs. C_50/50_) as separate problems. However, our paradigm addresses this by presenting probe trials less frequently than standard trials (one per eight trials versus six per eight trials). It therefore seems likely that learning primarily takes place during the standard A vs. B trials. Learning on probe trials is also impeded by the probabilistic nature of the feedback on these trials. The congruent effects of quinpirole at 0.25 mg/kg on negative probe trials in the VPVD task and *α*_loss_ in the PRL task supports the notion that performance on the probe trials reflects the estimated value of the positive and negative stimuli.

### Conclusion

We used two different approaches and tasks to study how wins and losses shape choice behaviour in reversal learning in the rat. The first approach employed a behavioural probe during a standard visual discrimination reversal task, while the second involved computational modelling to define learning rates and other latent factors underlying choice behaviour in a PRL task. We report that the D2-like receptor agonist quinpirole has profound and remarkably similar effects across the two tasks: a complete blockade of learning from losses. These findings extend previous work in rodents, non-human primates, and humans, and is relevant for human disorders in which cognitive flexibility is impaired, such as schizophrenia and Parkinson’s disease.

## Electronic supplementary material

ESM 1 (DOCX 519 kb)
