# Supplementary material for: Dopamine D2-like receptor stimulation blocks negative feedback in visual and spatial reversal learning in the rat: behavioural and computational evidence
Source: Psychopharmacology (Berl). 2019 Jun 19;236(8):2307–23. doi: 10.1007/s00213-019-05296-y (PMC6695374; doi:10.1007/s00213-019-05296-y)
Supplement: Supplementary file 1 — (DOCX 519 kb) [file 213_2019_5296_MOESM1_ESM.docx]

Electronic Supplementary Material pertaining to

**Dopamine D2-like receptor stimulation blocks negative feedback in visual and spatial reversal learning in the rat: behavioural and computational evidence**

Johan Alsiö^1†^, Benjamin U. Phillips^1,2^, Júlia Sala‑Bayo^1^, Simon R.O. Nilsson^1,3,4^, Teresa C. Calafat‑Pla^1^, Arazo Rizwand^1^, Jessica M. Plumbridge^1^, Laura López‑Cruz^1^, Jeffrey W. Dalley^1,5^, Rudolf N. Cardinal^1,5,6^, Adam C. Mar^1,3,4^, Trevor W. Robbins^1^

^1^Department of Psychology and Behavioural and Clinical Neuroscience Institute, University of Cambridge, United Kingdom; ^2^Department of Physiology, Development and Neuroscience, University of Cambridge, United Kingdom; ^3^Neuroscience Institute, New York University Medical Center, New York, NY, USA; ^4^Department of Neuroscience and Physiology, School of Medicine, New York University, New York, NY, USA; ^5^Department of Psychiatry, University of Cambridge, Cambridge, United Kingdom; ^6^Cambridgeshire & Peterborough NHS Foundation Trust, Box 190 (Liaison Psychiatry), Cambridge Biomedical Campus, Cambridge, UK;

**Supplementary methods**

*Touchscreen serial visual reversal (TSVR) task*

The procedure for this task has been previously described in detail (Alsiö et al. 2015). Briefly, as per the visual discrimination phase of the valence-probe task, rats were required to discriminate between two stimuli presented simultaneously on the screen (CS+ vs. CS-). If the rat touched the CS+, the tray light turned on a 45 mg precision pellet was delivered. If instead the CS- was touched, the house light turned on for a time-out of 5 seconds. Inter-trial interval was 5 seconds. Sessions ended after rats had completed 250 trials or earned 150 rewards, or after 60 minutes had passed, whichever occurred first. The running learning criterion was set 24 correct in 30 trials at least once during a session (this did not terminate the session). A retention session was included the day after rats initially reached criterion. In addition, another retention session was then included before the contingencies were reversed. This training protocol (retention 🡪 reversal until criterion🡪retention) then continued until the rats were able to reach criterion on the third day after any reversal; they were also required to complete at least 200 trials on the first day of reversal. See also Supplementary Table 1 and Supplementary Figure 1a.

*Statistical analysis of data from the TSVR task*

Data from the TSVR task were divided into separate phases depending on the performance of rats during running blocks of 30 trials (Alsiö et al. 2015). Only data up to (and including) the first block of 30 trials where a rat reached criterion (24 correct) were analysed. Trials were divided into ‘Early’, in which the rats had less than 11 correct trials in a running block of 30 trials, and ‘Late’ if the rats scored higher than 19 correct in any block of 30 trials; all other trials were treated as ‘Mid’. The number of errors and omissions in each phase were calculated and square-root transformed. Repeated-measures ANOVA were then performed with two within-subject factors: phase (3 levels) and dose (4 levels). Response and reward-collection latencies (in milliseconds) were log transformed and, for each rat, averaged across all analysed trials; latency data were then analysed using repeated-measures ANOVA with Dose (4 levels) as the within-subject variable. The Greenhouse-Geisser correction was employed when prompted by significant Mauchly’s tests of sphericity. Auxiliary measures were latencies to respond and to collect the reward.

Supplementary Table 1. Criteria for pre-training and testing stages in behavioural tasks

| Task/phase | Max trials | Max rewards | Limited hold | Criterion |
| --- | --- | --- | --- | --- |
| *Touchscreen serial visual reversal learning (TSVR)* | | | | |
| Stage01-03 | 100 | 100 | n/a | 100 rewards |
| Stage04 | 100 | 100 | 10 seconds | 80 rewards in two consecutive sessions |
| Stage05 | 100 | 100 | 10 seconds | 80 rewards in two consecutive sessions |
| Visual discrimination | 250 | 150 | 10 seconds | 24 correct in 30 trials running criterion |
| Serial reversal learning pre-training | 250 | 150 | 10 seconds | 24 correct in 30 trials running criterion; continue training until criterion is reached within 3 days after reversal (inclusive) |
| Serial reversal learning testing | 250 | 150 | 10 seconds | 24 correct in 30 trials running criterion |
|  |  |  |  |  |
| *Valence-probe visual discrimination reversal learning (VPVD)* | | | | |
| Stage01-03 | 100 | 100 | n/a | 100 rewards |
| Stage04 | 100 | 100 | 10 seconds | 80 rewards in two consecutive sessions |
| Stage05 | 100 | 100 | 10 seconds | 80 rewards in two consecutive sessions |
| Visual discrimination | 250 | 150 | 10 seconds | 24 correct in 30 trials running criterion |
| Pre-training reversal | 250 | 150 | 10 seconds | 24 correct in 30 trials running criterion |
| Pre-training VPVD | 200 | (200) | n/a | Minimum 5 days; additional days until 80% on standard trials |
| Novel stimuli VPVD | 200 | (200) | n/a | Minimum 5 days; additional days until 80% on standard trials |
| VPVD reversal learning | 200 | (200) | n/a | Minimum 10 or 14 days |
| *Serial spatial probabilistic reversal learning (PRL)* | | | | |
| Habituation | n/a | n/a | n/a | 1 session only |
| Conditioning | 100 | 100 | 30 seconds, after which 1 reward is given | 1 session only |
| Must Touch | 100 | 100 | n/a | 100 rewards |
| Must Initiate | 100 | 100 | n/a | 100 rewards |
| Full task baseline | 200 | (200) | n/a | 4 reversals/session |
| Full task test | 200 | (200) | n/a | One session/dose |

Across all training phases, where relevant, time-out on non-rewarded trials and inter-trial intervals were both set to 5 seconds. Numbers within brackets indicate it is impossible for the rats to reach the maximum number of rewards, due to the probabilistic nature of the reinforcement schedule.


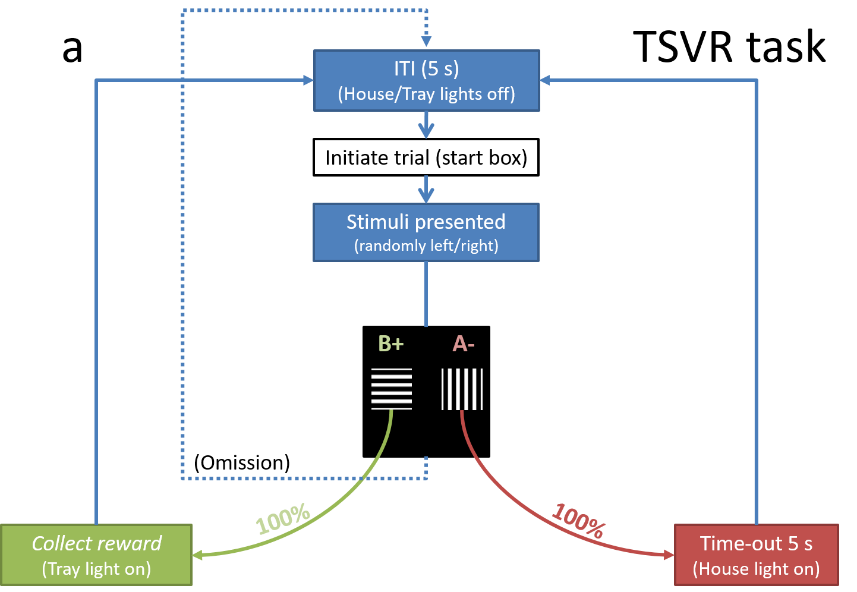

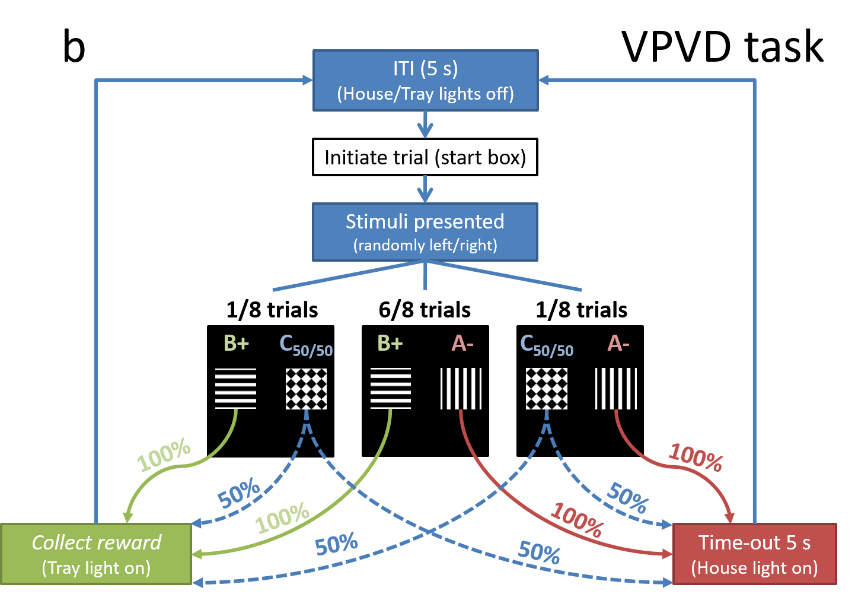

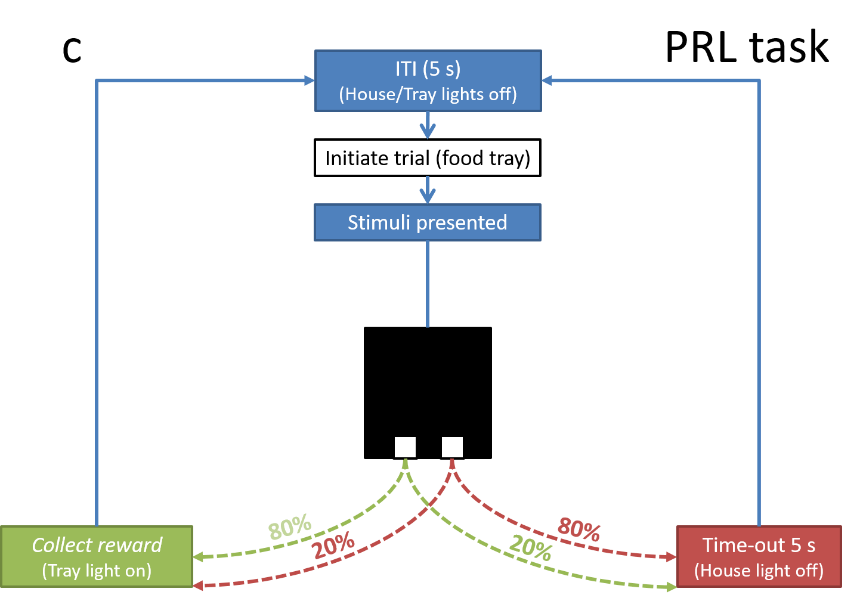


*Supplementary Figure 1*. Flowchart schematics of the trial structure in the a) touchscreen serial visual reversal task (TSVR); b) valence-probe visual discrimination reversal task (VPVD); and c) probabilistic reversal task (PRL). Percentages (dashed lines) represent reward probabilities.

**Computational analysis of serial probabilistic reversal learning data**

*(a) Reinforcement learning algorithm*

In the winning model **(model 2),** rewarded outcomes resulted in a higher value (V) for the stimulus (i) that was selected. The rate of this change was determined by the learning rate for wins *α_win_* via *V_i,t_*_+1_ ← *V_i,t_* + *α_win_*(*R_t_* – *V_i,t_*). In this equation the prediction error is represented by (*R_t_* – *V_i,t_*) with *R_t_*  giving the reward on trial *t*. In the case of nonrewarded trials, the value of *V_i_* decreased in accordance with *α_loss_* via *V_i,t_*_+1_ ← *V_i,t_* + *α_loss_*(*R_t_* – *V_i,t_*) when *R_t_=0.*  The impact of reward history on choice was given by *Q^reinf^_i,t_ = βV_i,t_* , in which *β* is a softmax inverse temperature coefficient reflecting the tendency of reward history to influence choices compared to other factors.

Side stickiness was given by *Q_l,t_* = *τL_l,t_*_–1_ where *L_l,t_*_–1_ reflects the subject’s location choice on the previous trial (1 if location *l* was chosen on the previous trial, or 0 otherwise) and *τ* is the side stickiness parameter, governing the degree to which side stickiness (perseveration to a location) influences choice.

Final action tendencies for each side per trial were determined by *Q_t_* = *Q_reinf,t_* + *Q_l,t_*, combining reinforcement learning with side stickiness*.* The probability of selecting each action on each trial was calculated via a softmax function:

$$P\left( \text{action}_{a} \right)=\text{softmax}^{a}\left( Q_{1}\text{...}Q_{n} \right)=\frac{e^{\text{Q}_{a}}}{\sum_{\text{k=}1}^{n} e^{\text{Q}_{k}}}$$

The final softmax probability values for each trial were subsequently compared with the empirical choices on each trial.

**Model 1** was identical to Model 2 except that the side stickiness component was not used.

**Model 3** was identical to Model 2 except that a single learning rate *α_rate_* was used instead of the dual learning rates *α_win_* and *α_loss_*.

**Model 4** was the experience-weighted attraction (EWA) model (Camerer & Ho 1999; den Ouden et al. 2013). Its parameters were *ρ*, the experience decay factor; *φ*, the decay factor for previous payoffs (equivalent to a learning rate); and *β*, a softmax inverse temperature governing action selection. For details, see den Ouden et al. (2013).

*(b) Hierarchical Bayesian model fitting and parameter estimation*

Free parameters from reinforcement learning algorithms were estimated using a hierarchical Bayesian approach. This approach produces a posterior distribution for all parameters of interest.

We defined prior distributions for all parameters. The parameters *α_rate_*, *α_win_*, *α_loss_*, *ρ*, and *φ*, which have the range [0, 1], were given a prior Beta(1.2, 1.2) distribution (den Ouden 2013). Reinforcement sensitivity, *β*, was given a prior Gamma(4.82, 0.88) distribution (Gershman 2016). Stickiness, *τ*, was given a Normal(0, 1) prior; perseverative parameters can be negative, indicating anti-perseveration (switching behaviour) (Christakou et al. 2013).

For each parameter we defined a separate distribution for each drug dose. These were the primary measures of interest.

Intersubject variability was assumed to be normally distributed with a mean of zero. Thus, for a given parameter, an individual subject’s parameter for a given session was calculated by combining (adding) the parameter value for the current relevant dose, with that subject’s (fixed) deviation from the group mean. The standard deviation of intersubject variability for each parameter (necessarily positive) was drawn as follows. For *α_rate_*, *α_win_*, *α_loss_*, *τ*, *ρ*, and *φ*, it was drawn from a prior half-normal(0, 0.05) distribution. For *β*, it was drawn from a prior half-normal(0, 1) distribution.

Final parameters were bounded as follows: *α* ∈ [0,1]; *β* ∈ [0,+∞]; *τ* ∈ [–∞, +∞].

These final parameters were then used in a reinforcement learning model, as described above, whose output was the probability of selecting each of the two actions on any given trial. The model was fitted (yielding posterior distributions for each parameter) by fitting these probabilities (arbitrarily, the probability of choosing the right-hand response) to actual choices (did the subject choose the right-hand response?).

We conducted the Bayesian analysis in RStan (Carpenter et al. 2017), which uses a Hamiltonian Markov chain Monte Carlo method to sample from posterior distributions of parameters. Primary values of interests were dose parameters and differences between them. The 95% highest posterior density interval (HDI) was used to describe posterior distributions. Given the assumptions (priors, model) and data, there is a 95% probability that the true value lies within the 95% HDI.

*(c) Model selection*

The winning model was selected via bridge sampling (see main text), which estimates the marginal likelihood of each model. This is the probability of the observed data given the model of interest, which encompasses both the probability of the data given specific values of the model’s parameters (goodness of fit) and the probability of the parameter values given the model (encapsulating a penalty for over-complex models; Occam’s razor). Given priors, and the assumption that the family of models represents all possible models of interest, the marginal likelihood also enables calculation of the posterior probability of each model. All models were given equal prior probability.

*Supplementary Table 2.* Model comparison for the four fitted models. The winning model (model 2) is shown in bold.

| **Model** | **Parameters** | **log marginal likelihood** | **log posterior P(model)** |
| --- | --- | --- | --- |
| Model 1 | *α_win_, α_loss_,* β | -4930.134 | -34.93359 |
| **Model 2** | ***α_win_, α_loss_,* β*,* τ** | **-4355.200** | **0.0000** |
| Model 3 | *α_rate_,* β*,* τ | -4398.240 | -43.04031 |
| Model 4 | *ρ, φ, β* | -4425.410 | -70.20952 |

**Supplementary Results**

*Effects of systemic D1- and D2-receptor antagonism on touchscreen serial visual reversal learning*

Thirty-two rats were trained on the serial visual reversal task until they were able to complete a reversal in 3 days or less. The animals were matched for baseline performance and randomised to receive either the dopamine D1-like receptor antagonist SCH39166 or the D2-like receptor antagonist Raclopride. Each rat received their allocated drug at three different doses plus vehicle in a Latin square design across 4 reversals. The data were split into learning phases for analysis (i.e. early, mid and late, reflecting a bias towards the previous CS+, no bias, and a bias towards the new CS+, respectively).

The impact of SCH39166 on errors in each phase of the reversal task was evaluated with a two-way ANOVA with Dose (4 levels) and Phase (3 levels) as within-subjects factors. The Greenhouse-Geisser correction was applied, since Mauchly’s test of sphericity was significant. There was no main effect of Dose (*F*_2.18,32.8_<1; NS) and no Dose x Phase interaction (*F*_3.11,46.6_<1; NS), but a main effect of Phase (*F*_1.34,20.1_=21.8; *p*<0.001) corresponding to the low number of errors during the late phase. Latencies to respond and to collect the reward were analysed separately using one-way ANOVA (latencies were collapsed across phases). SCH39166 significantly affected latency to respond (*F*_3,45_=3.72; *p*=0.016); Post-hoc analysis (Fisher’s LSD) revealed that the highest dose (0.1 mg/kg) slowed down responses (Table 2). SCH39166 also had a significant impact on latency to collect rewards (*F*_3,45_=7.05; *p*=0.001); *post-hoc* analysis indicated that the 0.1 mg/kg dose increased collection latency (Table 2).

The effect of raclopride on the number of errors committed in each phase of the serial reversal task was analysed with a two-way ANOVA with Dose (4 levels) and Phase (3 levels). The Greenhouse-Geisser correction was applied, since Mauchly’s test of sphericity was significant. There was no main effect of Drug (*F*_2.14,32.1_=1.50; NS) and no Drug x Phase interaction (*F*_3.23,48.4_<1; NS), but a significant main effect of Phase (*F*_1.26,18.8_=26.5; *p*<0.001). The effect of raclopride on latencies was tested in two separate one-way ANOVA with Dose as a within-subjects factor (4 levels) for responses and reward collection. Raclopride treatment significantly affected both response latency (*F*_3,45_=4.45; *p*=0.008) and reward collection latency (*F*_3,45_=7.59; *p*<0.001). Post-hoc analyses (Fisher’s LSD) showed that the highest dose (0.06 mg/kg) had increased choice latencies and that both the 0.03 and 0.06 doses significantly increased collection latencies (Table 2).


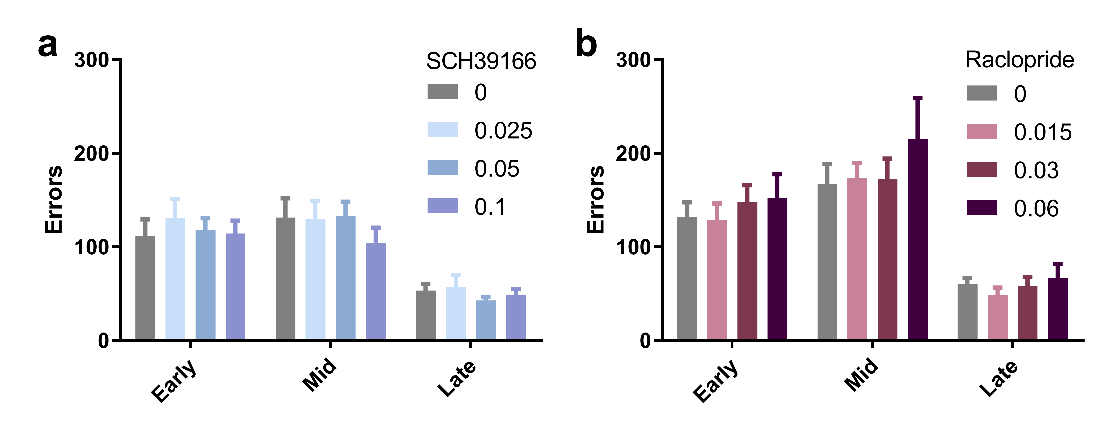


*Supplementary Figure 2*. Serial visual reversal performance after a) SCH39166 (n=16) or b) raclopride (n=16) treatment. Neither drug had a significant impact on the numbers of errors committed across the early, mid, or late phases of the task. Bars show mean±SEM for each dose and phase. Doses are in mg/kg.

**References**

Alsiö J., Nilsson S.R., Gastambide F., Wang R.A., Dam S.A., Mar A.C., Tricklebank M., Robbins T.W. (2015) The role of 5-HT2C receptors in touchscreen visual reversal learning in the rat: a cross-site study. Psychopharmacology (Berl). **232**:4017-31.

Camerer, C. and Ho, T.H. (1999) Experience‐weighted attraction learning in normal form games. Econometrica **67:** 827–874.

Carpenter, B., Gelman, A., Hoffman, M.D., Lee, D., Goodrich, B., Betancourt, M., Brubaker, M., Guo, J., Li, P. and Riddell, A. (2017) Stan: a probabilistic programming language. Journal of Statistical Software **76**: 1–32.

Christakou, A., Gershman, S.J., Niv, Y., Simmons, A., Brammer, M. and Rubia, K. (2013). Neural and psychological maturation of decision-making in adolescence and young adulthood. Journal of Cognitive Neuroscience **25**: 1807–23.

den Ouden, H.E.M., Daw, N.D., Fernandez, G., Elshout, J.A., Rijpkema, M., Hoogman, M., Franke, B. and Cools, R. (2013) Dissociable effects of dopamine and serotonin on reversal learning. Neuron **80:** 1090–1100.

Gershman, S.J. (2016) Empirical priors for reinforcement learning models. Journal of Mathematical Psychology **71**: 1–6.
